# Expression level of human TLR4 rather than sequence is the key determinant of LPS responsiveness

**DOI:** 10.1371/journal.pone.0186308

**Published:** 2017-10-11

**Authors:** Adeline M. Hajjar, Robert K. Ernst, Jaehun Yi, Cathy S. Yam, Samuel I. Miller

**Affiliations:** 1 Department of Comparative Medicine, University of Washington, Seattle, Washington, United States of America; 2 Department of Microbial Pathogenesis, University of Maryland, Baltimore, Maryland, United States of America; 3 Departments of Medicine, Microbiology and Genome Sciences, University of Washington, Seattle, Washington, United States of America; Northwestern University Feinberg School of Medicine, UNITED STATES

## Abstract

To address the role of Toll-like receptor 4 (TLR4) single nucleotide polymorphisms (SNP) in lipopolysaccharide (LPS) recognition, we generated mice that differed only in the sequence of TLR4. We used a bacterial artificial chromosome (BAC) transgenic approach and TLR4/MD-2 knockout mice to specifically examine the role of human TLR4 variants in recognition of LPS. Using *in vitro* and *in vivo* assays we found that the expression level rather than the sequence of TLR4 played a larger role in recognition of LPS, especially hypoacylated LPS.

## Introduction

Two coding human Toll-like receptor 4 (TLR4) single nucleotide polymorphisms (SNP; rs4986790 and rs4986791) resulting in Asp→Gly or Thr→Ile substitutions at amino acids 299 and 399, respectively, have been described [1]. In Europeans, the allele frequency of the double TLR4^D299G+T399I^ allele is ~7% [2]. The earliest study on these coding TLR4 SNPs suggested that they resulted in decreased responsiveness to inhaled lipopolysaccharide (LPS) [1]. Another report demonstrated prevalence of the single TLR4^D299G^ polymorphism in Africa (5–9% allele frequency), whereas the TLR4^D299G+T399I^ allele was found to be prevalent in Europe (3–9% allele frequency) [3]. The authors proposed that the TLR4^D299G^ variant initially arose to protect against cerebral malaria in Africa and that this allele was hyperresponsive to LPS, in contrast to the previous study. They proposed that the compensatory T399I mutation that reduced LPS responsiveness arose in Europe to protect from sepsis. That study also showed that the single TLR4^T399I^ variant is very rare. Other *in vitro* studies also demonstrated reduced responsiveness of TLR4^D299G+T399I^ to hexa- or penta-acylated LPS [4, 5].

Multiple association studies have linked these SNPs to a wide variety of diseases (early studies reviewed in [6]). Published reports describe association with sepsis, meningococcal disease, cerebral malaria, Crohn’s disease, ulcerative colitis, respiratory syncytial virus, atherosclerosis, rheumatoid arthritis, malignant melanoma, and metabolic syndrome, although others show no association for the same diseases suggesting complex genetic and epigenetic interactions [1, 4, 7–17]. Two additional reports suggested that the TLR4^D299G^ variant is protective against increased systolic blood pressure with increased obesity as well as against periodontitis, though both failed to examine the co-segregating rs4986791 SNP and thus actual genotype cannot be determined [18, 19]. Since the data in the literature is association data and even this data in some instances is incomplete and conflicting, we sought to develop a model system that would more clearly isolate the role of the coding TLR4 SNPs in response to LPS and disease states.

Thanks to the success in generating mice that display human-like responses to LPS through the expression of human TLR4 and MD-2 from human genomic BACs [20], we generated transgenic mice expressing either the TLR4^D299G^ or the TLR4^D299G+T399I^ human allele and compared them to mice expressing human TLR4^WT^. Although our comparisons were limited by the copy number of lines randomly generated, we found that the expression level of human TLR4 was the largest determinant of LPS response and that the actual sequence of human TLR4 did not reveal obvious differences in LPS recognition.

## Materials and methods

### Reagents

Ultrapure O111:B4 EC LPS was purchased from InvivoGen (San Diego, CA), and ODN1826 from Coley Pharmaceuticals (prior to acquisition by Pfizer, Düsseldorf, Germany). Monophosphorylated lipid A (MPL) was purchased from Avanti Polar Lipids Inc (Alabaster, AL). Antibodies for flow cytometry were purchased from Becton Dickinson (Franklin Lakes, NJ), eBioscience (San Diego, CA) or BioLegend (San Diego, CA). Anti-huTLR4 clones HTA125 and TF901 as well as anti-muTLR4 clone MTS510 were used for surface expression analysis.

### PA and YP LPS preparation

#### Bacterial growth conditions and strains

The strains used in this study are *P*. *aeruginosa* (PAO-1) and *Y*. *pestis* (KIM6-). *P*. *aeruginosa* was grown in Lysogeny broth (LB) supplemented with 1mM MgCl_2_ at 37°C with shaking and *Y*. *pestis* was grown in brain heart infusion (BHI) supplemented with 1mM MgCl_2_ at 37°C with shaking.

#### LPS and lipid A isolation

LPS was extracted by the hot phenol/water method [21]. Freeze-dried bacterial pellets were resuspended in endotoxin-free water at a concentration of 10 mg/ml. A volume of 12.5 ml of 90% phenol (Fisher Scientific, Pittsburgh, PA) was added and the resultant mixture was vortexed and incubated for 60 minutes in a hybridization oven at 65°C. The mixture was cooled on ice and centrifuged at 12,096 x *g* at room temperature for 30 minutes. The aqueous phase was collected and an equal volume of endotoxin-free water was added to the organic phase. The extraction was repeated and aqueous phases were combined and dialyzed against Milli-Q purified water to remove residual phenol and then freeze-dried. The resultant pellet was resuspended at a concentration of 10 mg/ml in endotoxin-free water and treated with DNase (Qiagen, Venlo, Limburg) at 100 μg/ml and RNase A (Qiagen, Venlo, Limburg) at 25 μg/ml and incubated at 37°C for 1 hour in a water bath. Proteinase K (Qiagen, Venlo, Limburg) was added to a final concentration of 100 μg/ml and incubated for 1 hour in a 37°C water bath [22]. The solution was then extracted with an equal volume of water-saturated phenol. The aqueous phase was collected and dialyzed against Milli-Q purified water and freeze-dried as above. The LPS was further purified by the addition of chloroform/methanol 2:1 [vol:vol] to remove membrane phospholipids [23] and further purified by an additional water-saturated phenol extraction and 75% ethanol precipitation to remove contaminating lipoproteins [24]. For Mass Spectrometry (MS) structural analysis 1 mg of purified LPS was converted to lipid A by mild-acid hydrolysis with 1% sodium dodecyl sulfate (SDS) (Sigma, St Louis, MO) at pH 4.5 as described previously [25].

#### MALDI-TOF mass spectrometry

Lipid A isolated by small-scale lipid A isolation procedures was analyzed on a AutoFlex Speed MALDI TOF mass spectrometer (Bruker Daltonics, Billerica, MA). Data was acquired in reflectron negative and positive modes with a Smartbeam laser with 1 kHz repetition rate and up to 500 shots were accumulated for each spectrum. Instrument calibration and all other tuning parameters were optimized using Agilent Tuning mix (Agilent Technologies, Foster City, CA). Data was acquired and processed using flexControl and flexAnalysis version 3.3 (Bruker Daltonics, Billerica, MA).

### Mice

#### Ethics statement

This study was carried out in strict accordance with the recommendations in the Guide for the Care and Use of Laboratory Animals of the National Institutes of Health. All protocols were approved by the Institutional Animal Care and Use Committee of the University of Washington.

#### Husbandry

All mice were bred in-house at the University of Washington. C57BL6/J mice were originally obtained from the Jackson Laboratory (Sacramento, CA). Mice were maintained in a specific pathogen free (SPF) facility in standard ventilated cages on autoclaved corn cob bedding with an automatic watering system and fed irradiated standard rodent chow with a 12 hr on/12 hr off light cycle. Several methods of euthanasia were used, depending on experimental manipulation. Mice were euthanized by CO_2_ narcosis, followed by a secondary method, when only bones were collected. However, for experiments using splenocytes (including those where bones were also collected), mice were euthanized by sharp cervical dislocation. Finally, for experiments where mice had received intraperitoneal LPS prior to euthanasia, mice were injected IP with an overdose of Beuthanasia-D (Schering-Plough Animal Health Corp., Union, NJ).

#### Generation

Human genomic BAC RP11-150L1 was modified by introducing point mutations using lambda Red mediated recombination and *galK* positive/negative selection to generate the D299G and T399I variants as described [26]. Primers used for recombineering are shown in Table 1. The entire TLR4 coding region was sequenced in the modified BACs prior to injection into fertilized B6C3 x B6 oocytes by the Transgenic Core at the University of Washington. Founders were backcrossed to B6 mice for 5 generations followed by backcrossing to TLR4/MD-2 DKO mice on a C57BL/6J background for at least another 5 generations. Throughout the figures and text, KO or TLR4 KO refers to a functional TLR4 KO, i.e. it is a TLR4/MD-2 DKO mouse that also expresses human MD-2, but not human TLR4. Only males are used in these studies because the human MD-2 BAC is integrated on the Y-chromosome limiting our ability to study females [20].

**Table 1:** Primer sequences.

oligo	sequence 5'-3'	info
307	TTTGACCATTGAAGAATTCCGATTAGCATACTTAGACTACTACCTCGATG-galK (CCTGTTGACAATTAATCATCGGCA)	5' oligo D299G
308	CCAGGGAAAATGAAGAAACATTTGTCAAACAATTAAATAAGTCAATAATA-galK (TCAGCACTGTCCTGCTCCTT)	3' oligo D299G
309	TTTGACCATTGAAGAATTCCGATTAGCATACTTAGACTACTACCTCGATG**G**TATTATTGACTTATTTAATTGTTTGACAAATGTTTCTTCATTTTCCCTG	D299G sense
310	CAGGGAAAATGAAGAAACATTTGTCAAACAATTAAATAAGTCAATAATA**C**CATCGAGGTAGTAGTCTAAGTATGCTAATCGGAATTCTTCAATGGTCAAA	D299G antisense
311	AAATGGCTTGAGTTTCAAAGGTTGCTGTTCTCAAAGTGATTTTGGGACAA-galK (CCTGTTGACAATTAATCATCGGCA)	5' oligo T399I
312	AACTCATGGTAATAACACCATTGAAGCTCAGATCTAAATACTTTAGGCTG-galK (TCAGCACTGTCCTGCTCCTT)	3' oligo T399I
313	AAATGGCTTGAGTTTCAAAGGTTGCTGTTCTCAAAGTGATTTTGGGACAA**T**CAGCCTAAAGTATTTAGATCTGAGCTTCAATGGTGTTATTACCATGAGT	T399I sense
314	ACTCATGGTAATAACACCATTGAAGCTCAGATCTAAATACTTTAGGCTG**A**TTGTCCCAAAATCACTTTGAGAACAGCAACCTTTGAAACTCAAGCCATTT	T399I antisense

#### BAC copy number

Copy number of the TLR4 BAC was determined using real-time PCR as previously described [20]. Primers used for amplification of TLR4 amplified both mouse and human TLR4 with equivalent efficiency allowing us to use a WT B6 mouse as a calibrator (copy number = 2) in the Pfaffl method to calculate the relative copy number of the transgene [27].

### Flow cytometry

Bone-marrow-derived macrophages (BMDM) were generated and stained as described for surface TLR4 expression using anti-human TLR4 clones HTA125 and TF901 and anti-mouse TLR4 clone MTS510 and appropriate isotype controls (mouse IgG2a and IgG1, and rat IgG2a respectively) [20]. Whole splenocyte stimulation and intracellular cytokine analysis was performed as described [20] with macrophage/monocyte population identified as CD11b+ CD11c low/negative (neg) CD3 neg CD19 neg cells.

### RT-PCR

Relative TLR4 expression levels were determined by real-time PCR as described [20] using an ABI 7300 Real Time PCR System and the Brilliant II SYBR Green QPCR reagents (Agilent Technologies Inc., Santa Clara, CA). Total RNA was isolated from cells or tissues using Trizol, DNase treated, and reverse transcribed using oligo dT priming and Superscript II (Invitrogen, Carlsbad, CA). A no-RT control confirmed RNA amplification. ß-actin was used to normalize the data between samples.

### Luminex

Serum samples were filtered in a 96-well 1.2 μm membrane HTS plate (Millipore, Billerica, MA) and diluted 1:4 or 1:12 in serum diluent. Cytokines (IL-1ß, IL-6, IL-10, IL-12p40, IL-12p70, TNFα) were quantified using a Bio-Plex 200 system with Bio-Plex Pro Assay Kits (Bio-Rad, Hercules, CA) following the manufacturer’s instructions as described [20].

### Statistics

Prism software (GraphPad, La Jolla, CA) was used for all statistical analyses. Multiple comparisons were performed using 1-way ANOVA followed by Bonferroni’s Multiple Comparison test.

## Results

### Generation of human TLR4 SNP mice

Initially, we modified the human TLR4 BAC to introduce the D299G mutation using BAC recombineering methods [26]. We then further modified this BAC to introduce the T399I mutation and generate the double D299G/T399I allele (often labeled as 399 in the figures). The BACs were then injected into fertilized oocytes at the University of Washington Transgenic Core (http://depts.washington.edu/compmed/transgenic/). Chimeric pups were identified by coat color and backcrossed to B6 mice and subsequently to TLR4/MD-2 double-KO mice, such that mice differed only in the sequence of human TLR4. The mice were also bred to humanized MD-2 mice [20]. Thus, in addition to human TLR4, these mice also express human MD-2 and lack mouse TLR4 and mouse MD-2. We generated and further characterized two lines for each TLR4 allele.

### Expression of human TLR4 variants

**Fig 1:**
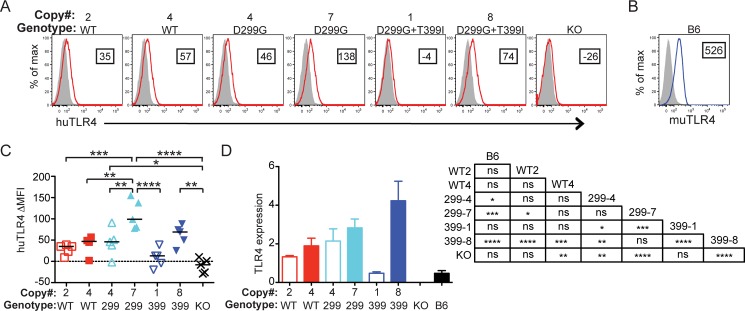
TLR4 expression correlates with copy number. (A) BMDM from each of the genotypes indicated above plots were stained with anti-huTLR4 clone HTA125 or isotype control. Boxed number in each plot shows ΔMFI of TLR4 (red histogram for huTLR4, blue histogram in B for muTLR4) vs. isotype control (filled grey histogram). (B) B6 mouse BMDM stained with anti-muTLR4. (C) Each symbol represents a separate BMDM preparation. All cells express huMD-2; red squares depict huTLR4^WT^, light blue triangles huTLR4^D299G^ (299), and dark blue inverted triangles huTLR4^D299G+T399I^ (399). X (KO) does not express TLR4. Open symbols have lower copy numbers than closed symbols. Brackets show significant pair-wise comparisons using 1-way ANOVA followed by Bonferroni’s Multiple Comparison test. (D) Relative TLR4 mRNA expression in BMDM from each genotype, including WT B6 BMDM (average of 3 separate BMDM preparations). The same primers were used for human and mouse TLR4, and expression relative to ß-actin is shown. Plotted are the means +/- SD. 1-way ANOVA results for D are shown to the right. *P<0.05, **P<0.01, ***P<0.001, ****P<0.0001, ns = not significant.

We first examined copy number using methods as described in [20] and found that our new lines consisted of 4 and 7 copies of huTLR4^D299G^ and 1 and 8 copies of huTLR4^D299G+T399I^, as compared to our previously generated 2- and 4-copy lines of huTLR4^WT^. While increasing BAC copy number from 1 to >48 correlates with increased expression (we did not have lines with identical copy numbers for comparison), when independent lines of same copy number were examined differences in staining intensity could be seen [28]. Therefore, we next examined expression of TLR4 in bone-marrow derived macrophages (BMDM) by staining for surface expression (Fig 1A–1C) and by RNA expression (Fig 1D). Increased copy number clearly resulted in increased RNA (Fig 1D) although protein expression appears to plateau by 7 copies, with no increase in the 8-copy line (Fig 1A and 1C). For this analysis, we also stained cells with a second clone (TF901; S1 Fig) that confirmed our results with clone HTA125, due to potential differences between antibodies directed at human TLR4 [29].

### LPS responsiveness in primary splenic macrophages/monocytes

**Fig 2:**
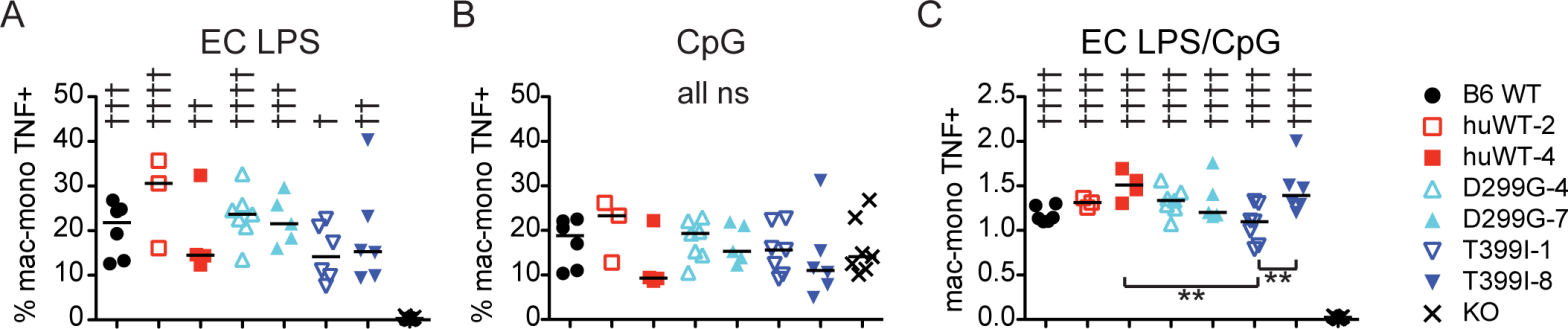
All TLR4 SNP primary splenocyte macrophage/monocyte populations respond to EC LPS as measured by intracellular cytokine staining. (A) Percent of mac/mono cells that are TNF+ in response to 1000 ng/ml EC LPS or (B) 10 μg/ml CpG. (C) Percent of mac/mono TNF+ in response to EC LPS from (A) divided by the percent TNF+ in response to CpG from (B). † above genotype show results compared to KO. † *P*<0.05, †† *P*<0.01, ††† *P*<0.001, †††† *P*<0.0001, ns = not significant. Brackets show significant pairwise comparisons. Combined data from 7 experiments with each symbol representing a separate experiment for each genotype. Note that not each genotype was tested in each experiment. huWT = huTLR4^WT^, D299G = huTLR4^D299G^, T399I = huTLR4^D299G+T399I^, number after dash shows copy number of huTLR4 transgene.

To determine whether we had restored *E*. *coli* (EC) LPS responsiveness in the splenic macrophage/monocyte population expressing the variant huTLR4 alleles, we used primary splenocyte intracellular cytokine staining (Fig 2) as described [20, 30]. An example of the gating strategy and TNF production in the macrophage/monocyte population is shown in S2 and S3 Figs. Compared to the percentage of cells producing TNF in TLR4 KO splenocytes, all humanized TLR4 lines showed an increase in TNF+ cells following EC LPS stimulation (Fig 2A). The absolute percent positive varied from day to day, as did the response to the positive control CpG (Fig 2B; each symbol is from a different mouse spleen on a different day). Therefore, we normalized the results by dividing the percentage of TNF+ cells in response to LPS by the percent TNF+ in response to CpG (Fig 2C). This correction helped reveal decreased TNF production by the 1-copy compared to 8-copy TLR4^D299G+T399I^ line.

**Fig 3:**
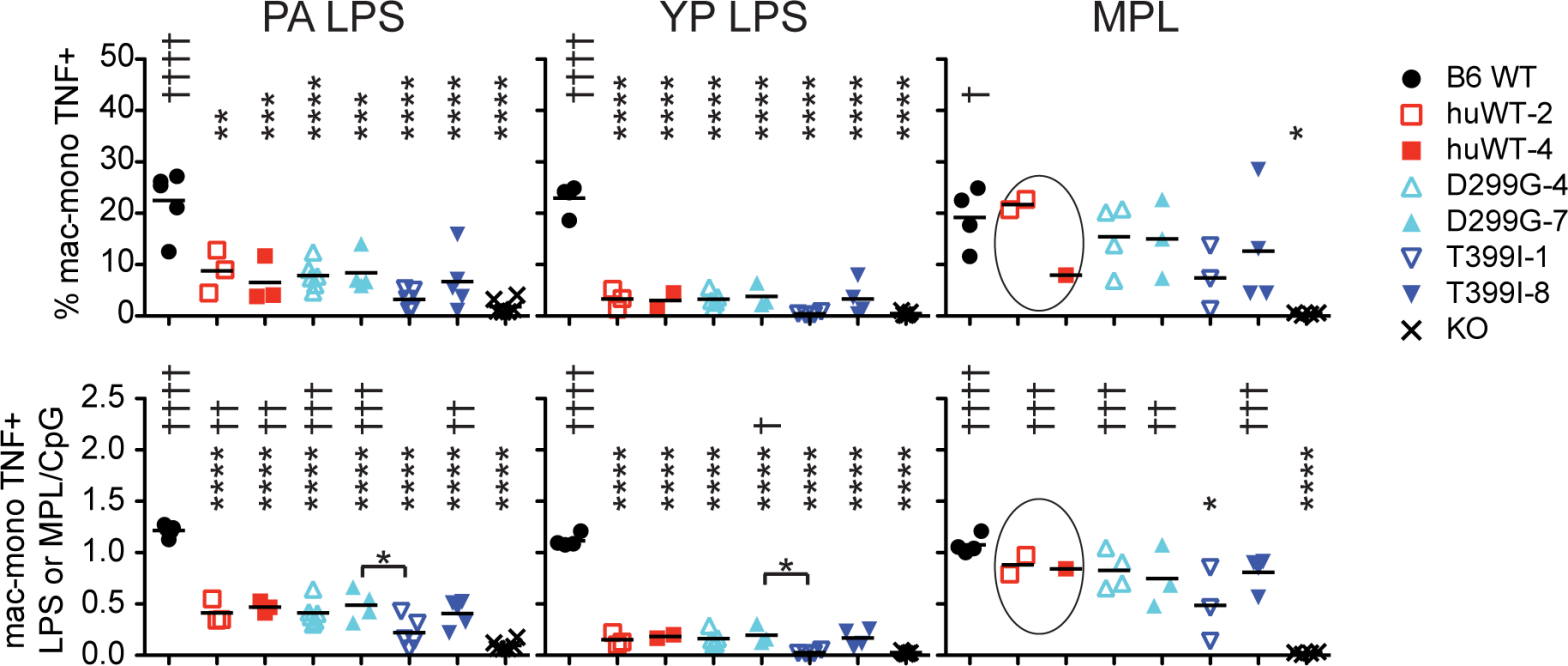
Reduced responsiveness to hypoacylated LPS by TLR4 SNP primary splenocyte mac/mono populations. Percent of mac/mono cells that are TNF+ in response to 1000 ng/ml PA LPS, YP LPS or MPL, top row, or percent TNF+ corrected by the percent TNF+ in response to CpG, bottom row. * above genotypes shows results compared to B6 whereas † is compared to KO. Data for huTLR4^WT^ in response to MPL were combined for 1-way ANOVA since the 4-copy line was only tested once and our previous studies did not reveal any differences between the 2- and 4-copy lines.

We also examined the primary splenic responses to hypoacylated LPS or monophosphorylated lipid A (MPL; Fig 3). We used a penta-acylated preparation of *Pseudomonas aeruginosa* (PA) LPS [31], as well as tetra-acylated *Yersinia pestis* (YP) LPS [20]. Unlike the response to EC LPS, all humanized TLR4 mice, including huTLR4^WT^, showed reduced activity in response to hypoacylated LPS, as compared to WT B6 mice. In addition, following correction by the response to CpG, all lines except the 1-copy TLR4^D299G+T399I^ line showed significantly increased response to PA LPS or MPL compared to the KO responses. The 1-copy TLR4^D299G+T399I^ line did not respond to either PA or YP LPS even though we observed a response to EC LPS (Fig 2).

### *In vitro* cytokine responses

**Fig 4:**
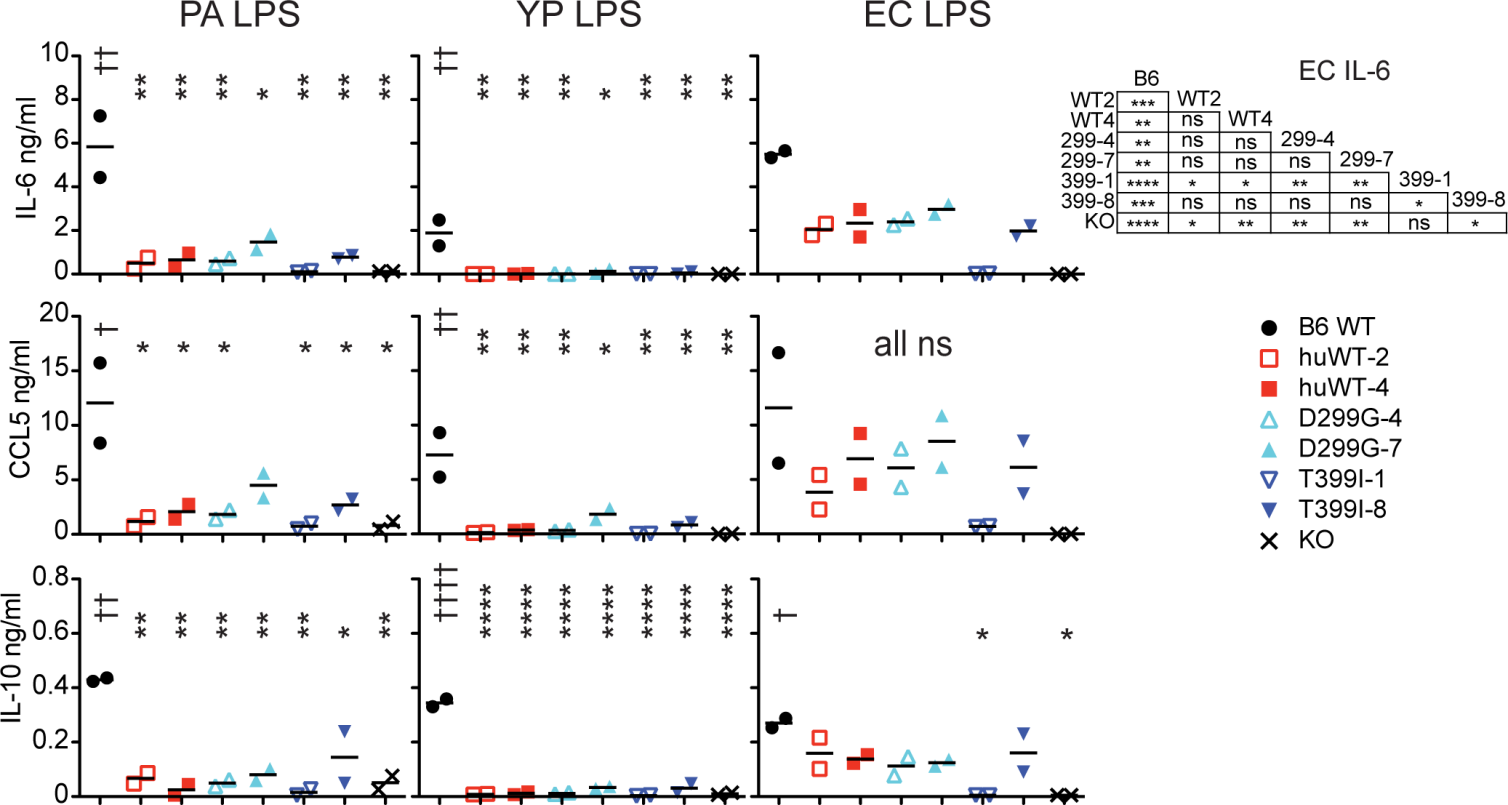
Cytokine responses in supernatants from BMDM stimulated with indicated LPS. An equal number (10^6^ cells) of BMDM were plated in 96-well plates and stimulated with 1000 ng/ml PA or YP LPS, or 10 ng/ml EC LPS. Cytokines secreted into the supernatants were measured 24-hr later by Luminex. Results from 2 independent BMDM preparations are shown. * above genotypes shows results compared to B6 whereas † is compared to KO. 1-way ANOVA results for the EC IL-6 responses are shown in the table.

We next generated bone-marrow-derived macrophages (BMDM), and stimulated cells with either PA, YP LPS, or EC LPS, and measured IL-6, CCL5, and IL-10 cytokines in the supernatants 24 hr later by Luminex (Fig 4). All humanized BMDM secreted very low levels of cytokines in response to PA or YP LPS, in contrast to WT B6 cells. In fact, the responses were not significantly different from TLR4 KO responses, although there was a trend towards higher values in the 7-copy TLR4^D299G^ and 8-copy TLR4^D299G+T399I^ lines compared to lower copy numbers. All humanized lines and the KO line were significantly reduced for IL-6 production, as compared to WT B6 cells, when stimulated with EC LPS. However, all humanized lines with the exception of the 1-copy TLR4^D299G+T399I^ line had increased IL-6 production compared to the KO. In contrast to the previous assays, the 1-copy TLR4^D299G+T399I^ line did not respond to EC LPS.

### *In vivo* cytokine responses

**Fig 5:**
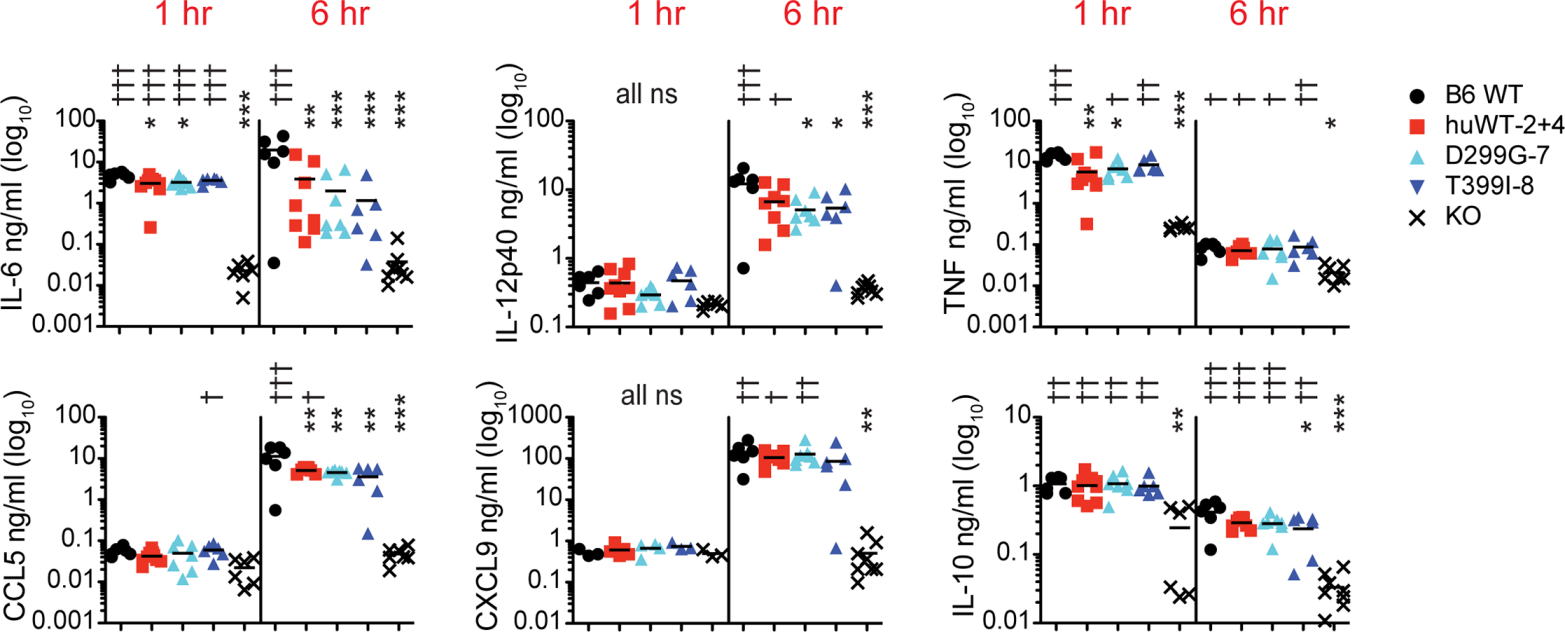
Serum cytokine responses to 100 μg EC LPS 1 + 6 hr post IP injection. Mice of the indicated genotypes were injected IP with EC LPS and then bled from the retro-orbital sinus 1 hr after injection. At 6 hr, the mice were euthanized for a terminal sample collection. Shown are combined data from 3 experiments, with 1 experiment containing 1 and 6 hr time points whereas the other 2 had either the 1 or 6 hr time point. All genotypes were included in all experiments. Data are plotted on a log scale for easier visualization due to large variation. Line indicates mean for genotype. * above symbols shows results compared to B6 whereas † is compared to KO.

**Fig 6:**
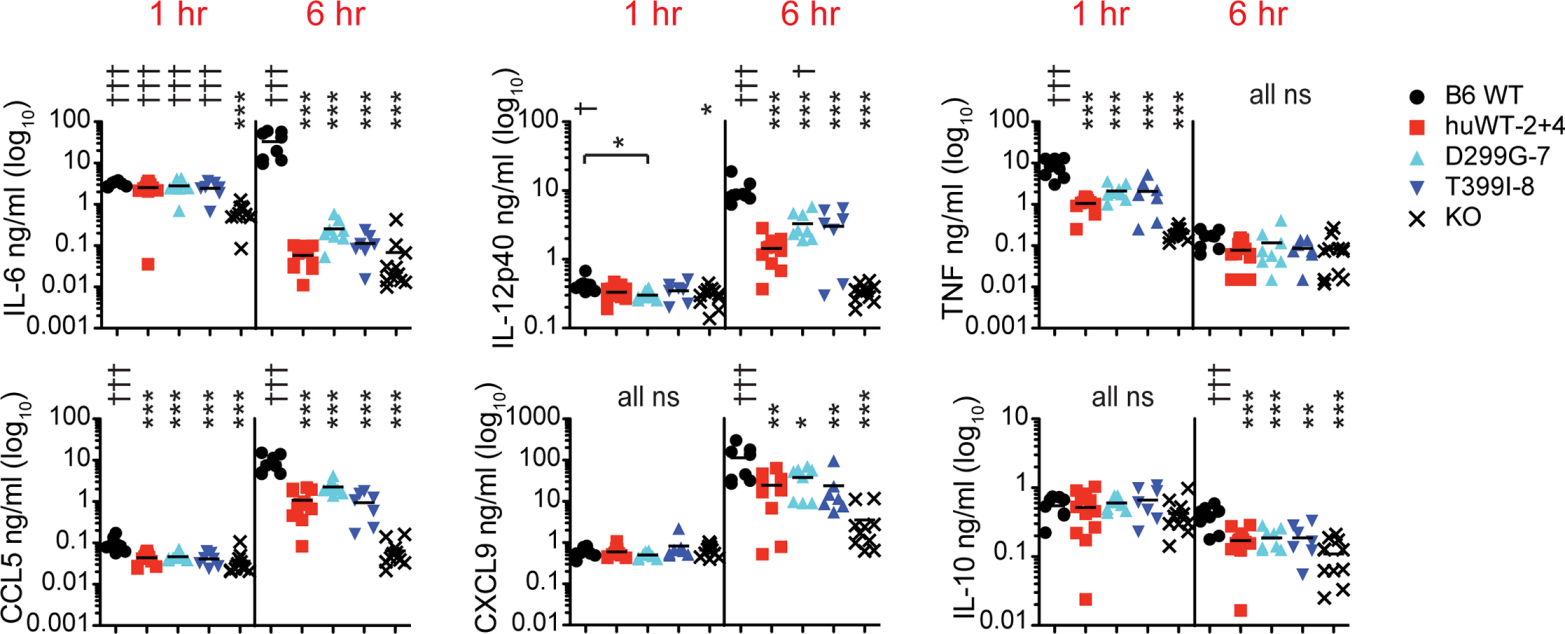
Serum cytokine responses to 50 μg PA LPS 1 + 6 hr post IP injection. Mice of the indicated genotypes were injected IP with PA LPS and then bled from the retro-orbital sinus 1 hr after injection. At 6 hr, the mice were euthanized for a terminal sample collection. Shown are combined data from 2 experiments. Data are plotted on a log scale for easier visualization due to large variation. * above symbols shows results for that genotype compared to B6 whereas † is compared to KO.

To determine whether differential recognition of hypoacylated LPS could be measured *in vivo*, we extended our studies into mice, and injected mice intraperitoneally (IP) with either EC or PA LPS (Fig 5 and Fig 6). In response to EC LPS, IL-6 and TNF were increased at 1 hr and decreased by 6 hr, although B6 maintained high IL-6 levels at 6 hr (Fig 5). IL-10 was also increased at 1 hr in all genotypes compared to KO though absolute levels were vey low. At 6 hr, IL-12p40, CCL5 and CXCL9 were all increased. However, in all cases no significant differences were observed between the humanized lines indicating that the sequence of TLR4 did not alter responses to EC LPS.

Mice were also injected with hypoacylated PA LPS (Fig 6). When comparing the values of IL-6 at 1 hr to the EC response, it is obvious that PA LPS induced less IL-6 at 1 hr, although a small response was also observed in KO mice, potentially indicating that non-TLR4 contaminants were present in the preparation despite repurification [32]. By 6 hr, the levels of IL-6 were comparable between PA and EC LPS in B6 mice; however, the humanized TLR4 lines all had reduced levels that were not significantly different from KO mice. There is a trend, however, towards higher levels in the TLR4 SNP mice compared to human TLR4^WT^ for both IL-6 and IL-12p40 at 6 hr, though the results are not significant.

## Discussion

There is a large body of literature that continues to expand examining the association of TLR4 SNPs with disease. However, no study has clearly, reproducibly, and mechanistically linked the common coding TLR4 SNPs (rs4986790 and rs4986791) with disease. TLR4 and MD-2 are coreceptors that bind directly to the lipid A component of LPS [33, 34]. The structure of lipid A varies between bacterial species and is also regulated in response to culture/environmental conditions or infection of a host [35, 36]. Highly endotoxic lipid A found in *E*. *coli* and *Salmonella* is predominantly hexa-acylated and bisphosphorylated. This lipid A can be detoxified by the removal of one phosphate resulting in mono-phosphoryl lipid A (MPLA), a known vaccine adjuvant that results in a selective decrease in MyD88-dependent responses while TRIF-dependent responses are maintained [37, 38]. These results suggest that the structure of the lipid A molecule alters signaling. *Pseudomonas aeruginosa* contains lipid A that is penta-acylated although cystic fibrosis isolates produce hexa-acylated molecules [39]. Similarly, *Yersinia pestis* produces hexa-acylated lipid A at room-temp but a tetra-acylated molecule at 37°C [40, 41]. These hypoacylated molecules induce reduced inflammatory responses selectively through human TLR4/MD-2 compared to mouse TLR4/MD-2 again demonstrating that the structure of lipid A alters signaling through TLR4/MD-2 [20, 31]. Similarly, TLR4 and MD-2 modification (by targeted mutagenesis) alters recognition of lipid A [42].

The crystal structure of TLR4^D299G+T399I^ in complex with *E*. *coli* LPS demonstrated conformational changes at the site of the D299G polymorphism though not at the site of LPS binding [43]. One *in vitro* study that showed equivalent LPS binding between the D299G variant and WT TLR4 suggested that decreased inflammatory cytokine production mediated by huTLR4^D299G^ is due to decreased recruitment of MyD88 and TRIF [44]. Our studies here do not reveal any obvious mechanistic differences in natural LPS recognition, including hypoacylated LPS, by the TLR4 variants, although one study suggests that the TLR4^D299G+T399I^ variant is impaired in dimerization in response to MPLA [29]. In fact, our results confirm what others have clearly demonstrated *in vitro* and in mouse studies [45–48], that increased expression of TLR4 results in increased responsiveness to LPS. Our results also suggest that non-coding TLR4 SNPs (e.g. rs2770150, rs7873784, rs10759932) that could result in altered expression levels of TLR4 are likely more important in disease association than the coding SNPs.

## Supporting information

S1 Fig TLR4 expression using TF901 clone. (A) BMDM from each of the genotypes indicated above plots were stained with anti-huTLR4 clone TF901 or isotype control. Boxed number in each plot shows ΔMFI of TLR4 (red histogram for huTLR4, blue histogram in B for muTLR4) vs. isotype control (filled grey histogram). Histograms from 2 separate experiments are shown. (B) B6 mouse BMDM stained with anti-muTLR4. (C) Each symbol represents a separate BMDM preparation. All cells express huMD-2; red squares depict huTLR4WT, light blue triangles huTLR4D299G (299), and dark blue inverted triangles huTLR4D299G+T399I (399). X (KO) does not express TLR4. Open symbols have lower copy numbers than closed symbols. Brackets show significant pair-wise comparisons using 1-way ANOVA followed by Bonferroni’s Multiple Comparison test. *P<0.05.
(TIF)

S2 Fig Gating strategy to examine primary splenocyte responses. Data from a single experiment shown in Fig 2 are presented to demonstrate the gating strategy used to identify the macrophage/monocyte population as previously published (Ref 20).
(TIF)

S3 Fig TNF production in the macrophage/monocyte population from S2 Fig. TNF histograms of the macrophage/monocyte population stimulated with indicated ligands. The % of macrophages/monocytes producing TNF is shown in each histogram.
(TIF)
