# Supplementary figures and images for: Expression level of human TLR4 rather than sequence is the key determinant of LPS responsiveness
Source: PLoS One. 2017 Oct 11;12(10):e0186308. doi: 10.1371/journal.pone.0186308 (PMC5636155; doi:10.1371/journal.pone.0186308)

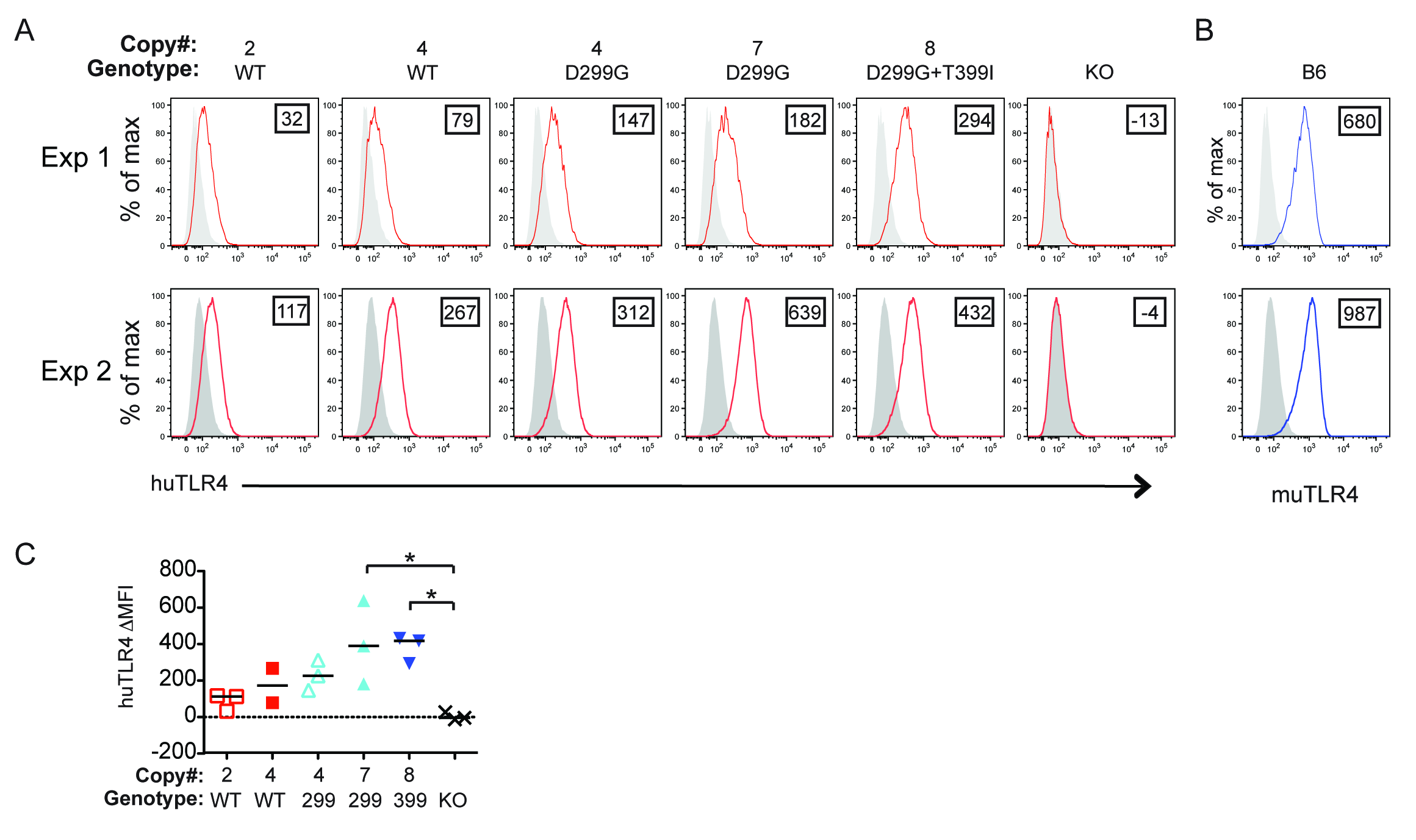

Supplement: S1 Fig — (A) BMDM from each of the genotypes indicated above plots were stained with anti-huTLR4 clone TF901 or isotype control. Boxed number in each plot shows ΔMFI of TLR4 (red histogram for huTLR4, blue histogram in B for muTLR4) vs. isotype control (filled grey histogram). Histograms from 2 separate experiments are shown. (B) B6 mouse BMDM stained with anti-muTLR4. (C) Each symbol represents a separate BMDM preparation. All cells express huMD-2; red squares depict huTLR4WT, light blue triangles huTLR4D299G (299), and dark blue inverted triangles huTLR4D299G+T399I (399). X (KO) does not express TLR4. Open symbols have lower copy numbers than closed symbols. Brackets show significant pair-wise comparisons using 1-way ANOVA followed by Bonferroni’s Multiple Comparison test. *P<0.05. (TIF) [file pone.0186308.s001.tif]

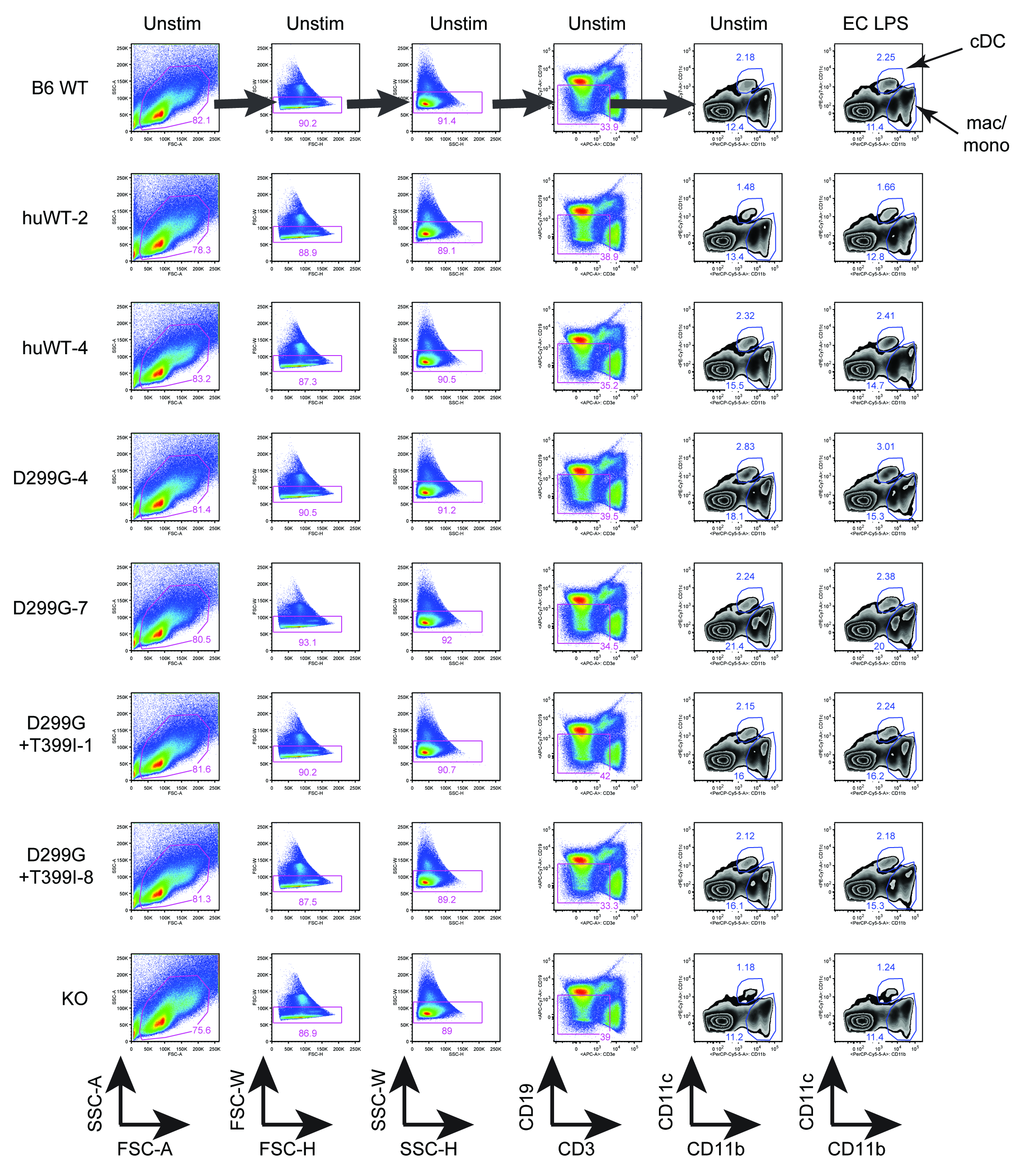

Supplement: S2 Fig — Data from a single experiment shown in Fig 2 are presented to demonstrate the gating strategy used to identify the macrophage/monocyte population as previously published (Ref 20). (TIF) [file pone.0186308.s002.tif]

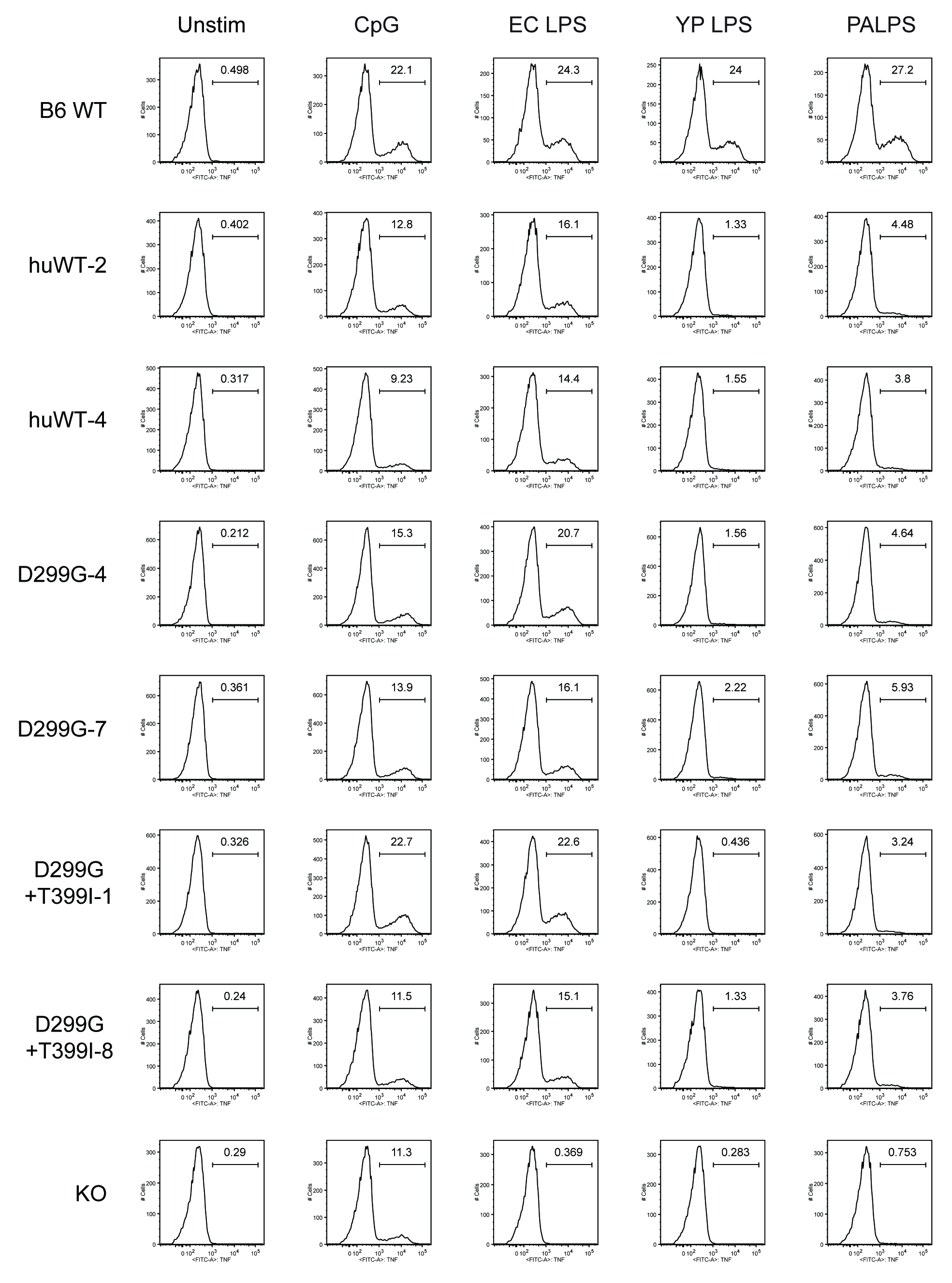

Supplement: S3 Fig — TNF histograms of the macrophage/monocyte population stimulated with indicated ligands. The % of macrophages/monocytes producing TNF is shown in each histogram. (TIF) [file pone.0186308.s003.tif]
